# Assessment of health promotion action for tuberculosis of end tuberculosis action plan (2019–2022) in China

**DOI:** 10.1186/s12889-024-19413-w

**Published:** 2024-07-30

**Authors:** Shuaihu Ni, Jia Wang, Xue Li, Wei Chen, Yan Qu, Yanlin Zhao, Xiaofeng Luo

**Affiliations:** 1 National Center for Tuberculosis Control and Prevention, Chinese Center for Disease Control and Prevention (CDC), Beijing, China; 2 School of Public Health, Lanzhou University, Lanzhou, Gansu China

**Keywords:** Tuberculosis, Action plan, Health promotion, Awareness, Health education

## Abstract

**Background:**

Tuberculosis is a chronic infectious disease that endangers people’s health, and China is a country with a high burden of tuberculosis. To accelerate the progress towards ending TB, the Chinese government implemented the End TB Action Plan (2019–2022), which consists of six actions. Among the End TB Action Plan, health promotion was conducted to improve the awareness of TB knowledge among Chinese people. The purpose of this study is to evaluate the effectiveness of implementing TB health promotion activities outlined in the End TB Action Plan, and to provide recommendations for future TB prevention and control plan.

**Methods:**

A cross-sectional study was conducted in nine Chinese provinces in 2022. A total of 11,920 Chinese people filled out the questionnaire. Logistic regression analysis was used to explore that overall awareness of TB core information is associated with whether they received TB health education.

**Results:**

The study involved 11,920 Chinese participants. The overall awareness rate of the core information of TB was 84.2%, which does not reach the 85% goal of the End TB Action Plan. The single awareness rate that TB is a chronic infectious disease and that the vast majority of TB patients can be cured were 75.3% and 76.0%. Participants who received TB health education had a higher awareness rate of TB core information. Participants who were elderly, retired or had a primary school education or below had poor awareness of the core information of TB. Participants who were elderly, lived in urban areas, were equipment operators or had a primary school education or below received less TB health education. Participants mainly received TB health education through TV (44.6%) and Internet (37.8%), preferred TV, broadcasts or movies (58.9%) and Internet advertising (54.7%). Participants preferred illustrated (46.2%) and audio-visual (44.8%) advertising materials. The common sources used to answer health-related questions on the internet were We-Medias (48.3%) and search engines (38.9%). Participants preferred to use short-form videos (66.8%) and illustrations (53.1%) to answer health-related questions.

**Conclusions:**

Health promotion action for TB had a positive effect in China, but the awareness of TB was inadequate among Chinese people, and it is necessary to strengthen TB health education for those people. Moreover, TB prevention and control institutions should advertise TB more in audio-visual and illustrated forms on the Internet and audio-visual media. Health-related questions should be published in short-form video and illustrated forms in the We-Media and search engine more.

## Background

Tuberculosis (TB) is a chronic infectious disease and about 1 billion people have died from TB over the past two centuries [1–3]. In 2022, there were 10.6 million new TB cases globally, with an incidence rate of 133 per 100,000 people [4]. The estimated number of new TB patients in China in 2022 was 748,000, and the estimated TB incidence was 52 per 100,000 [4]. Among the 30 countries with high TB burden, China had the third largest burden of TB [4].

The Chinese government conducted the national TB prevention and control plan to strengthen the prevention and control of TB [5, 6]. In the plan, health promotion is an used strategy to raise awareness of TB, which plays a very important role in achieving the goal of ending TB [7–9]. To achieve the global target of ending TB by 2030, in response to the Moscow Declaration, the National Health Commission of China cooperated with 7 departments to formulate the End TB Action Plan in 2019 [10–12]. The End TB Action Plan consists of six actions, of which health promotion action for TB is mainly aimed at improving awareness of TB among Chinese people [10]. The health promotion action for TB proposed to mobilize the participation of the whole society, carry out various forms of publicity activities and health education for Chinese people. The goal was for the public awareness rate of the core information of tuberculosis prevention and control to reach more than 85.0% [10].

The results of the 13th Five-Year Plan (2016–2020) showed that the public awareness rate of TB was 82.5%, but it can only reflect the public awareness of TB in 2020 and cannot reflect the current TB awareness of the Chinese people [6]. In the three years since the implementation of the Action Plan to End TB (2019–2022), China has continued to strengthen the prevention and control of TB, holding theme activities on March 24 every year, employing public figures to publicize TB information, carrying out various forms of TB awareness activities, and using new forms of media to educate the public about TB. Therefore, the objective of this study was to assess increased awareness, acceptance of TB health education, the need for TB health education sources and the need for health-related questions. This study not only shows the effect of the TB health promotion action but also shows the awareness, health education, and health-related questions of the Chinese people about TB in 2022. Meanwhile, the results of this study can also provide a basis for the next national TB prevention and control plan.

## Methods

### Study design

This study is a cross-sectional study based on face-to-face interviews conducted in 2022 in nine provincial-level administrative regions in eastern, central and western China. The eastern part is Guangdong Province, Zhejiang Province and Tianjin City. The central region is Anhui, Hubei and Shanxi provinces; The western region is Yunnan province, Chongqing city and Ningxia Province. Tianjin and Chongqing are municipalities directly under the central government, with the same administrative level as the provinces. The participants were permanent urban and rural residents aged 15 years and above (including non-local residents who lived in the survey place for more than 6 months).

### Survey methods and data collection

Multistage stratified cluster sampling was used in this study, and the sample size was calculated using the PASS [13]. The study assumes that the item with the lowest awareness rate of core information in 2022 is 60.0%, the relative error is 0.2, α is 0.05, and the number of respondents at each survey point is 100. The interclass correlation coefficient is 0.3, and the effective response rate is 85.0%. The final calculation results show that 1082 people need to be sampled in each province. To ensure accuracy, the sample size was increased to 1200 people. Each province set up 12 survey sites and each survey site had 100 people [14]. The provincial TB prevention and control institutions were responsible for sampling.

Each province had a total of 12 survey sites. The provincial TB prevention and control institutions queried the proportion of urban and rural registered populations in the province. The proportion of urban and rural survey sites was the same as the proportion of urban and rural registered people in the province. According to the population aged 15 and above in each township, the survey sites were selected according to the equal proportion probability sampling method. In these selected towns, streets and villages are also selected according to the equal proportion probability sampling method.

The county (district) TB prevention and control institutions determined the name list of all the people in the selected streets (villages) through the household registration records, and marked the name list of all the people aged 15 years and above. A simple random sampling method with replacement was used to select 100 subjects for investigation. Due to work, refusal to cooperate, illness and other reasons, if the selected subject could not be interviewed, new subjects would be selected.

Face-to-face interviews were adopted in this survey to ensure that the questionnaire was answered by the selected people and that the respondents could understand the meaning of the questions in the questionnaire. Interviews allowed people with visual impairments or lower literacy levels to participate in the survey. The interview began with the training of investigators from the county (district) TB prevention and control institutions. The investigators arranged a time and place, notified people who would be selected to fill out the questionnaire, and audio recordings were made throughout the process. Provincial and municipal TB prevention and control institutions carried out supervision and data quality spot checks at the investigation site. After the interview, the county (district) TB prevention and control institutions arranged for personnel to collect questionnaires and input questionnaire data in EpiData. The paper materials were kept in the county (district) TB prevention and control institutions. The data were used in this study after being reviewed by county (district), municipal and provincial TB control institutions and the Chinese Center for Disease Control and Prevention.

#### Questionnaire design

The questionnaire for this study was modified from the questionnaire on TB core information, compiled by the National Center for Tuberculosis Prevention and Control of the Chinese Center for Disease Control and Prevention. The questionnaire of this study is mainly divided into three parts: the basic situation of the survey participants, the core information of tuberculosis prevention and control, and the acceptance of health education. (1) The basic information of the study participants included gender, habitation, age, education, and occupation. (2) The awareness of the core information of TB prevention and control: Q1: TB is a chronic infectious disease; Q2: TB is mainly transmitted through the respiratory tract; Q3: Coughing and expectoration for more than 2 weeks should be suspected of TB; Q4: Not spitting, covering your mouth and nose when coughing or sneezing, and wearing a mask can all reduce the spread of TB; Q5: The vast majority of TB patients can be cured. (3) The status of receiving TB health education: whether you have received TB health education, the information delivery sources of TB health education, the preferred information delivery sources of TB health education, the types of materials to accept TB health education, the sources used to answer health-related questions on the internet, the preferred materials used to answer health-related questions. The information delivery sources refer to the way people receive TB health education, and the types of materials refer to different kinds of TB propaganda materials. For example, when publishing an article on TB health education on the network, the network is the information delivery source and the text is the types of material. In this study, search engines represent the websites that are indexed by search engines.

#### Quality control

The provincial TB prevention and control institutions were responsible for sampling and leadership, and county-level TB control institutions were responsible for organizing implementation. The county level tuberculosis prevention and control institutions were responsible for reviewing the survey data entered on the day, including checking the number of questionnaires, ensuring forms were fully completed, logicality, authenticity and correcting problems in time. Logicality means that the content of the questionnaire was not inconsistent. The contents of some questionnaires were unreasonable, for example, the occupation of young people was listed as retirement and these questionnaires were deleted. Authenticity means that the interview was not affected by others, and the interview was conducted by the selected person. For example, some people were unable or unwilling to the investigation site for face-to-face interviews, so the investigators contacted alternate participants to be interviewed. Meanwhile, the interviews were conducted in separate rooms and participants were not allowed to query the material in any way. During the investigation, the provincial and municipal tuberculosis prevention and control institutions carried out on-site supervision and spot checking of data quality. After the completion of the investigation, municipal tuberculosis prevention and control institutions verified the database, and created a summary for provincial tuberculosis prevention and control institutions. The provincial TB prevention and control institutions approved the report to the Chinese Center for Disease Control and Prevention, which conducted the final database data verification and summary. Some questionnaires were not filled out completely, and those with incomplete information were deleted during the audit.

### Statistical analysis

The number and percentage of demographic characteristics were analyzed in this study and used as descriptive statistics. The number and awareness rate were used to describe the awareness of the survey object, and the formula was as follows [15]:

$$\eqalign{& {\rm{The}}\,{\rm{overall}}\,{\rm{awareness}}\,{\rm{rate}}\,{\rm{of}}\,{\rm{TB}}\,{\rm{core}}\,{\rm{information}} \cr & = {\matrix{\sum {{\rm{The}}\,{\rm{number}}\,{\rm{of}}\,{\rm{TB}}\,{\rm{core}}} \hfill \cr {\rm{information}}\,{\rm{items}}\,{\rm{correctly}} \hfill \cr {\rm{answered}}\,{\rm{by}}\,{\rm{participants}} \hfill \cr} \over \matrix{{\rm{The}}\,{\rm{number}}\,{\rm{of}}\,{\rm{TB}}\,{\rm{core}} \hfill \cr {\rm{information}}\,{\rm{items}} \hfill \cr {\rm{answered}}\,{\rm{by}}\,{\rm{participants}} \hfill \cr} } \times 100\% \cr}$$

$$\eqalign{& {\rm{The}}\,{\rm{single}}\,{\rm{awareness}}\,{\rm{rate}}\,{\rm{of}}\,{\rm{TB}}\,{\rm{core}}\,{\rm{information}} \cr & = {\matrix{\sum {{\rm{The}}\,{\rm{number}}\,{\rm{of}}\,{\rm{single}}\,{\rm{TB}}\,{\rm{core}}} \hfill \cr {\rm{information}}\,{\rm{items}}\,{\rm{correctly}} \hfill \cr {\rm{answered}}\,{\rm{by}}\,{\rm{participants}} \hfill \cr} \over \matrix{{\rm{The}}\,{\rm{number}}\,{\rm{of}}\,{\rm{single}}\,{\rm{TB}}\,{\rm{core}} \hfill \cr {\rm{information}}\,{\rm{items}} \hfill \cr {\rm{answered}}\,{\rm{by}}\,{\rm{participants}} \hfill \cr} } \times 100\% \cr}$$

The bar chart was used to describe the information delivery sources for participants to receive tuberculosis health education, the preferred information delivery sources and materials of participants receiving TB health education, and the sources and preferred materials used to answer health-related questions on the Internet. The Chi-square test was used to compare the impact of TB health education on participants’ awareness of TB core information. In addition, a logistic regression analysis was performed to determine the factors influencing participants’ overall awareness of TB core information and participants’ acceptance of TB health education. In logistic regression analysis, crude odds ratios were obtained using univariate logistic regression; those with P-values less than 0.25 were considered significant, and multiple logistic regression was continued to obtain their adjusted odds ratios, with *P* ≤ 0.05 considered statistically significant [16, 17].

## Results

### Participant characteristics

There were 11,920 Chinese participants in this study, including 5841 males (49.0%) and 6079 females (51.0%). Many participants were aged 15–44 (39.2%), with only 26.1% of participants aged 60 and above. 46.1% of participants resided in cities, and 53.9% of participants resided in rural areas. Most participants had a junior high school degree, while those with a bachelor’s degree or above were the least (10.7%). The majority of participants were farmers (non-livestock and poultry breeders) (32.1%), with only 2.3% of participants being equipment operators. 80.3% of the participants had received TB health education, and 19.7% had not received TB health education (Table 1).

**Table 1:** Demographic characteristics of participants

Characteristics	Number	Percentage(%)
Sex		
Males	5841	49.0
Females	6079	51.0
Age		
15–44	4675	39.2
45–59	4136	34.7
60-	3109	26.1
Area residence		
Urban	5496	46.1
Rural	6424	53.9
Education		
Primary and below	3193	26.8
Junior middle school	4116	34.5
Senior middle school and equivalent education	1875	15.7
Junior college	1457	12.2
Bachelor and above	1279	10.7
Occupation		
Government	1201	10.1
Enterprise business owners	1367	11.5
Social services	1550	13.0
Students	490	4.1
Farmer (non-livestock and poultry breeders)	3826	32.1
Retired	1197	10.0
Farmers (livestock and poultry breeders)	1004	8.4
Equipment operator	278	2.3
Others	1007	8.5
Have received education about TB		
Yes	9575	80.3
No	2345	19.7

Social services: persons engaged in social and residential life services, including commercial staff such as sales and procurement, and other service staff (such as cleaning staff, barber, washing and darning staff, etc.). Non-livestock and poultry breeders: agricultural production personnel, forestry production and auxiliary personnel, etc

### Awareness of TB core information among all participants

The participants’ overall awareness rate about core TB information was 84.2%. However, only 55.3% of participants knew all TB core information, and 2.9% did not know any TB core information. Only 75.3% of the participants answered that TB is a chronic infectious disease correctly, and 76.0% of the participants answered that the vast majority of TB patients can be cured correctly (Table 2).

### Awareness of TB core information among participants who received TB health education

80.3% of the participants received tuberculosis health education, and their overall awareness rate of tuberculosis core information was 87.9%. The overall awareness rate of tuberculosis core information of participants who did not receive tuberculosis health education was 69.3%. Participants who received TB health education had a higher awareness rate of each TB core information question than participants who did not receive TB health education (*P* < 0.001). Among participants who received TB health education, awareness of the definition of TB (79.7%) and whether it was curable (79.2%) remained below 85.0% (Table 2).

**Table 2:** Awareness of TB core information among participants

Information	All participants *n* (%)	Received TB health education *n* (%)		χ^2^	*P*-value
		Yes	No		
TB is a chronic infectious disease	8977(75.3)	7633(79.7)	1344(57.3)	507.32	< 0.001
TB is mainly transmitted through the respiratory tract	10,986(92.2)	9185(95.9)	1801(76.8)	951.43	< 0.001
Coughing and expectoration for more than 2 weeks should be suspected of TB	10,934(91.7)	9109(95.1)	1825(77.8)	741.42	< 0.001
Not spitting, covering your mouth and nose when coughing or sneezing, and wearing a mask can all reduce the spread of TB	10,244(85.9)	8562(89.4)	1682(71.7)	486.55	< 0.001
The vast majority of TB patients can be cured	9059(76.0)	7583(79.2)	1476(62.9)	271.91	< 0.001
Overall	50,200(84.2)	42,072(87.9)	8128(69.3)	2440	< 0.001

N (%) awareness number and awareness rate of TB core information

### The association between overall awareness and participant characteristics

Participants’ overall awareness of the core information about TB was related to age, education, and occupation. Participants who were elderly, retired or had a primary school education or below had poor awareness of the core information of TB (Table 3).

**Table 3:** Factors associated with overall awareness of TB core information

Characteristic	Rate (%)	Unadjusted		Adjusted	
		COR (95%CI)	*P*-value	AOR (95%CI)	*P*-value
Sex					
Male	54.9	Ref		Ref	
Female	55.8	1.04(0.96–1.11)	0.33	-	-
Age					
15–44	60.1	Ref		Ref	
45–59	55.8	0.84(0.77–0.91)	< 0.001	0.90(0.82-1.00)	0.04
60-	52.5	0.60(0.55–0.66)	< 0.001	0.73(0.64–0.82)	< 0.001
Area residence					
Urban	55.6	Ref		Ref	
Rural	55.2	0.98(0.92–1.06)	0.68	-	-
Education					
Primary or below	50.6	Ref		Ref	
Junior middle school	55.2	1.21(1.10–1.32)	< 0.001	1.15(1.04–1.28)	0.006
Senior middle school or equivalent education	56.8	1.29(1.15–1.44)	< 0.001	1.21(1.06–1.38)	0.005
Junior college	60.9	1.52(1.34–1.73)	< 0.001	1.34(1.14–1.57)	< 0.001
Bachelor or above	59.3	1.43(1.25–1.63)	< 0.001	1.21(1.01–1.44)	0.04
Occupation					
Government	59.0	Ref		Ref	
Enterprise business owners	61.9	1.13(0.96–1.32)	0.14	1.16(0.99–1.37)	0.07
Social services	56.3	0.89(0.77–1.04)	0.15	0.94(0.80–1.11)	0.49
Students	61.8	1.12(0.91–1.40)	0.29	1.09(0.87–1.37)	0.44
Farmers (non-livestock and poultry breeders)	58.4	0.98(0.86–1.11)	0.72	1.25(1.06–1.48)	0.008
Retirement	42.4	0.51(0.43–0.60)	< 0.001	0.69(0.57–0.84)	< 0.001
Farmers (livestock and poultry breeders)	44.7	0.56(0.47–0.66)	< 0.001	0.71(0.58–0.86)	< 0.001
Equipment operators	51.8	0.75(0.57–0.97)	0.03	0.83(0.63–1.09)	0.18
Others	52.5	0.77(0.65–0.91)	0.002	0.89(0.74–1.07)	0.21

Rate: the rate of answering all TB core information correctly. COR: crude odds ratio. AOR: adjusted odds ratio. CI: confidence interval. Ref: Reference

### The association between receiving TB health education and participant characteristics

Participants who were elderly, urban residents, equipment operators or had a primary school education or below were less likely to receive TB health education (Table 4).

**Table 4:** Factors associated with whether participants received TB health education

Characteristic	Rate (%)	Unadjusted		Adjusted	
		COR (95%CI)	*P*-value	AOR (95%CI)	*P*-value
Sex					
Male	80.8	Ref		Ref	
Female	79.9	0.94(0.86–1.03)	0.21	0.97(0.88–1.06)	0.49
Age					
15–44	82.5	Ref		Ref	
45–59	80.1	0.85(0.76–0.95)	0.003	0.92(0.81–1.04)	0.19
60-	77.4	0.72(0.65–0.81)	< 0.001	0.84(0.74–1.01)	0.02
Area residence					
Urban	78.8	Ref		Ref	
Rural	81.7	1.20(1.10–1.31)	< 0.001	1.13(1.002–1.26)	0.02
Education					
Primary or below	75.3	Ref		Ref	
Junior middle school	82.2	1.52(1.35–1.70)	< 0.001	1.67 (1.48–1.90)	< 0.001
Senior middle school or equivalent education	82.6	1.56(1.35–1.80)	< 0.001	1.82(1.54–2.15)	< 0.001
Junior college	82.7	1.57(1.34–1.84)	< 0.001	1.81(1.49–2.20)	< 0.001
Bachelor or above	80.8	1.38(1.18–1.62)	< 0.001	1.43(1.15–1.78)	0.001
Occupation					
Government	86.8	Ref		Ref	
Enterprise business owners	75.9	0.48(0.39–0.59)	< 0.001	0.46 (0.37–0.57)	< 0.001
Social services	77.7	0.53(0.43–0.65)	< 0.001	0.50(0.40–0.63)	< 0.001
Students	91.6	1.66(1.17–2.41)	0.01	1.44(1.00-2.11)	0.05
Farmers (non-livestock and poultry breeders)	83.1	0.75(0.62–0.90)	< 0.001	0.91(0.72–1.14)	0.42
Retirement	76.7	0.50(0.40–0.62)	< 0.001	0.60(0.46–0.76)	< 0.001
Farmers (livestock and poultry breeders)	83.0	0.74(0.58–0.93)	0.01	0.89(0.68–1.16)	0.40
Equipment operators	71.9	0.39(0.29–0.53)	< 0.001	0.38(0.27–0.53)	< 0.001
Others	70.6	0.36(0.29–0.45)	< 0.001	0.41(0.32–0.52)	< 0.001

Rate: the rate of receiving TB health education. COR: crude odds ratio. AOR: adjusted odds ratio. CI: confidence interval. Ref: Reference

### Information delivery sources and materials to receive TB health education

**Fig. 1:**
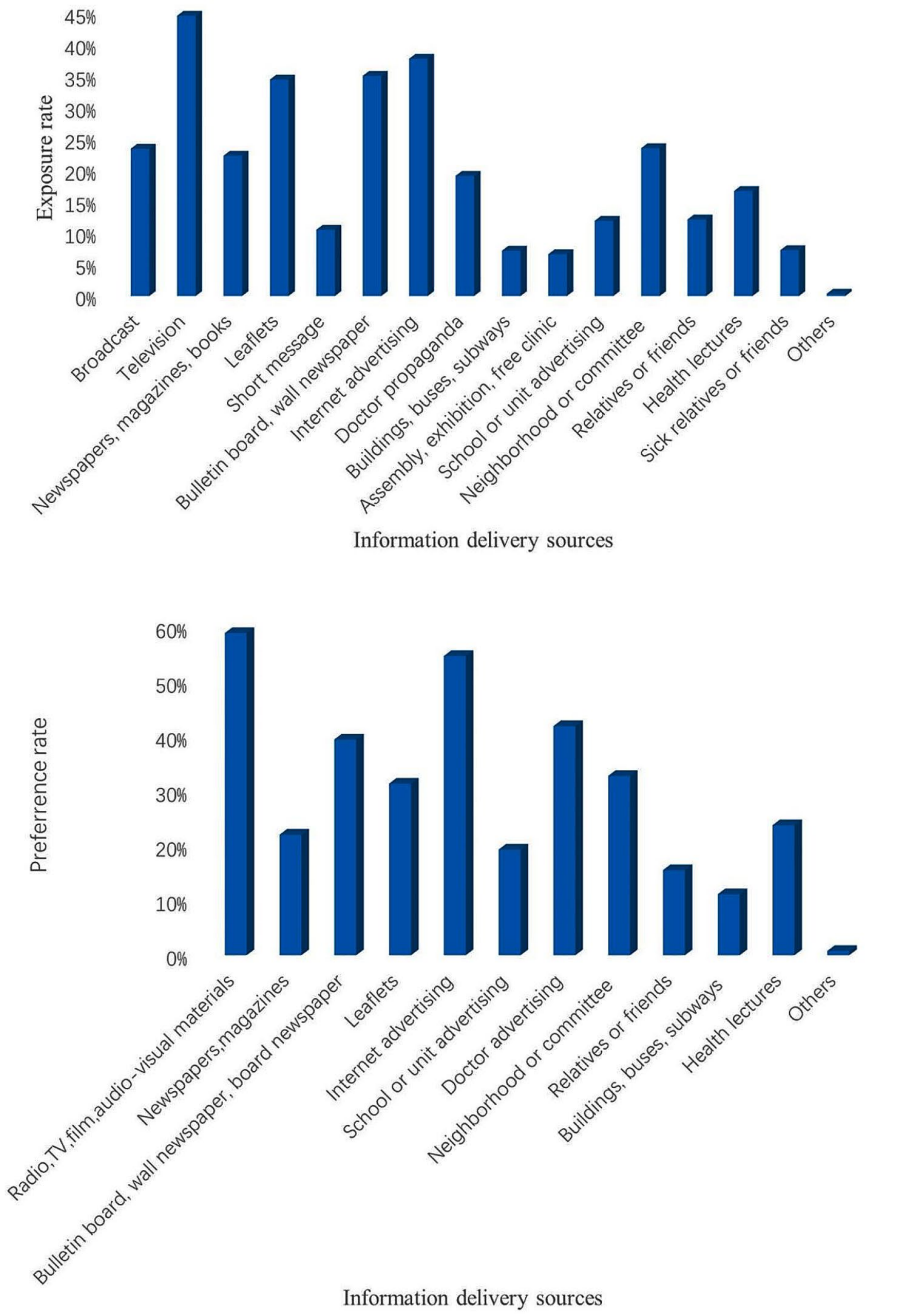
TB health education sources (**A**) used and (**B**) preferred. Exposure rate: The number of participants who exposed to tuberculosis health information divided by the number of all participants. Preference rate: The number of participants who preferred the information delivery sources of TB health education divided by the number of all participants

**Fig. 2:**
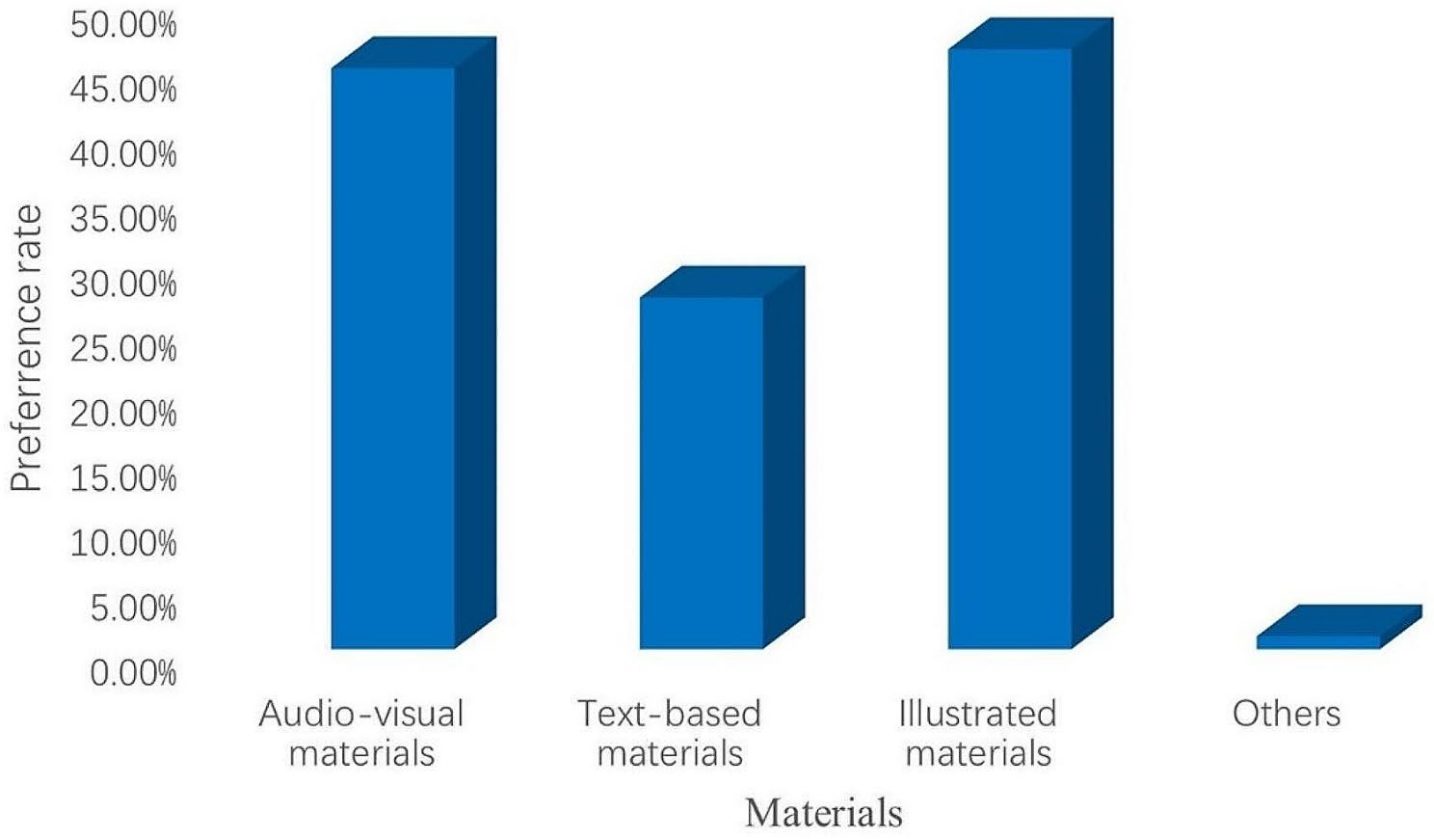
TB advertising materials preferred. Preference rate: The number of participants who preferred the TB advertising materials divided by the number of all participants

The common information delivery sources for participants to receive tuberculosis health education were TV (44.6%) and Internet advertising (37.8%) (Fig. 1). The main information delivery sources that participants preferred to use to receive TB health education were radio, TV, film, audio-visual medias (58.9%) and Internet advertising (54.7%) (Fig. 2). Participants were more willing to accept illustrated (46.2%) and audio-visual (44.8%) TB advertising materials (Fig. 2).

### The sources and preferred materials used to answer health-related questions on the internet

**Fig. 3:**
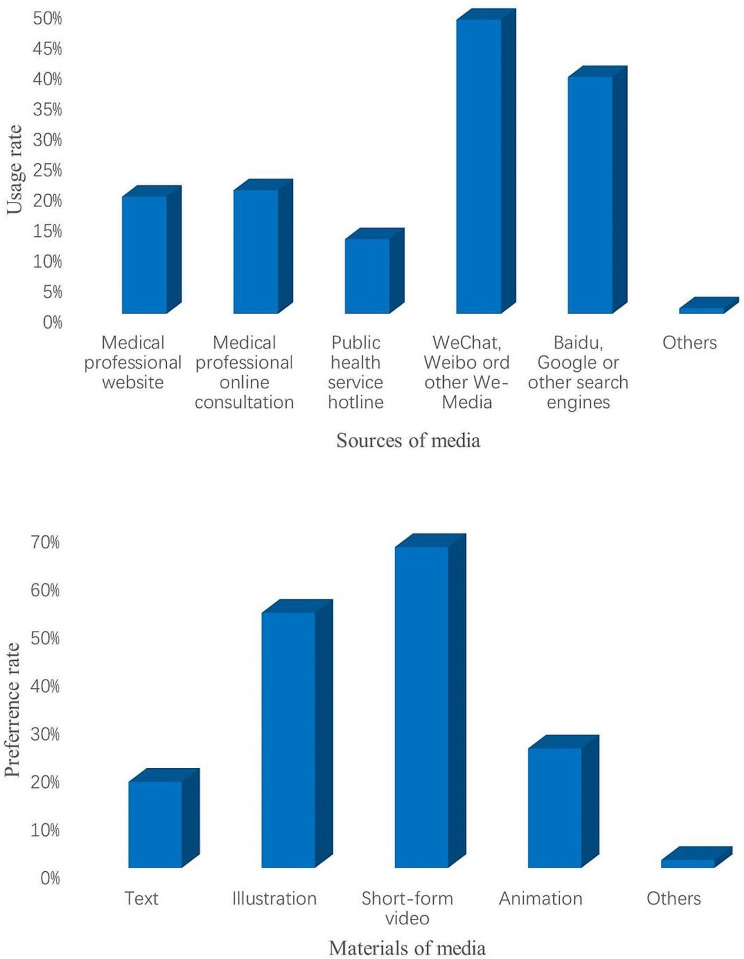
The sources (**A**) and preferred materials (**B**) used to answer health-related questions on the Internet. Usage rate: The number of participants who used sources to answer health-related questions divided by the number of all participants. Preference rate: The number of participants who preferred health-related questions materials divided by the number of all participants

The common sources used to answer health-related questions on the internet were WeChat, Weibo and other We-Medias (48.3%), and Baidu, Google and other search engines (38.9%) (Fig. 3). Participants preferred to use short-form video (66.8%) and illustration (53.1%) to answer health-related questions on the Internet (Fig. 3).

## Discussion

Although the annual incidence and death rate of TB in China is decreasing, China is a high burden country for TB [4]. At present, the progress of universal health coverage in China is slow, especially the treatment service capacity [18]. Good awareness of TB can help to control TB and relieve pressure on treatment services [19]. However, Chinese people’s awareness of TB was still not up to expectations [20–22].

In this study, the overall awareness rate of TB core information among the Chinese public is 84.2%. Compared with the 13th Five-Year Plan (2016–2020), the overall awareness rate of TB core information improved, but it is lower than 85.0%. It indicated that the implementation of the End TB action Plan improved the awareness of the Chinese people on tuberculosis but had not reached the ideal goal. In addition, the overall awareness rate of TB core information was 87.9% in people who received TB health education, which proved that TB health education played a positive role in increasing awareness of TB, and the public overall awareness rate of TB can reach the expectation through health education [23]. Therefore, in future work, TB prevention and control institutions in China need to continue to spread TB health education, popularize TB core information to the public and improve public awareness of TB.

Only 55.3% of the people knew all the core information about tuberculosis, which indicated that many people in China did not have a comprehensive understanding of TB and only knew part of the information about TB [15]. The awareness rate that tuberculosis is a chronic infectious disease was only 75.3%. The awareness rate of people who have received health education was lower than 85.0%, even lower than the 13th Five-Year Plan. The results showed that some people did not know that tuberculosis is an infectious disease. Some people thought that TB is a noncommunicable disease, leading to an easy neglect of TB prevention. Some people may not know they have TB, causing delays in treatment and continuing to spread it to others [20]. TB health education could not effectively improve the awareness of TB as a chronic infectious disease in China [24]. The awareness rate that the vast majority of TB patients could be cured was only 76.0%, and even among those who had received health education, the awareness rate was lower than 85.0%. This information has important implications for improving treatment confidence and compliance in TB patients, and even for preventing the occurrence of drug-resistant TB, but it’s that more is needed in order for it to work to the desired goal [22, 25]. Therefore, TB prevention and control institutions in China need to innovate the publicity methods of some TB information, focus on the weak points in the grasp of TB core information, and improve the Chinese people’s comprehensive understanding of TB prevention and control knowledge.

From the results of this study, older people received less TB health education and had worse awareness of the core messages of TB [26]. The elderly had insufficient knowledge about the harm of TB and were rarely willing to receive TB health education [27]. However, the elderly are at high risk of tuberculosis [28], so the elderly should be regarded as the key population to raise TB awareness in future work, and TB prevention and control institutions in China should choose a simple and clear form to carry out TB health education for the elderly. People of urban residence received less TB health education. In the past, rural areas had a high incidence of TB in China, but the End TB Action Plan designated rural areas as the focus of TB control and conducted more health promotion activities in rural areas, so that rural residents received more TB health education [10, 28]. In future work, TB prevention and control institutions in China can carry out more similar activities in both urban and rural areas, carry out more TB health education, and publicize more TB control measures. People with lower education level received less TB health education and had worse awareness of TB core information [15]. Schools are one of the key places for TB health education in China. Every year, schools are obliged to publicize TB knowledge to students. However, people with lower levels of education left school earlier, and thus had fewer opportunities to obtain TB health education and had less awareness of TB [29]. Therefore, TB prevention and control institutions in China need to provide more TB health education opportunities for people with low education levels. Health education on infectious diseases, including tuberculosis, should be carried out at all school ages, so that people at all levels of education can receive TB health education and improve their awareness of TB core information. Equipment operators and people in other occupations lacked the opportunity to receive TB health education, perhaps because of less TB health promotion in the workplace or long working hours. Retirees were unable to receive TB health education in the workplace or on campus, so equipment operators and people in other occupations received less TB health education and had poor cognition of TB [30]. Therefore, TB prevention and control institutions in China can organize TB health education activities for retirees, equipment operators and people in other occupations in their leisure time and hold TB health education classes to raise their awareness of TB [31].

Compared with the 13th Five-Year Plan, in the past, Chinese people mainly received TB health education through TV (57.6%) and leaflets (37.8%), whereas now, information is transmitted through the TV (44.6%) and Internet (37.8%). In the past, Chinese people mainly preferred Internet (55.8%) and audio-visual media (65.3%) such as radio and TV to receive TB health education, and now Chinese people also prefer the Internet (54.7%) and audio-visual media (58.9%). Due to the high popularity of traditional audio-visual media and the approval of the government, Chinese people have more trust in the information conveyed by traditional audio-visual media [32]. Because it is inconvenient to save, difficult to update, and difficult to understand, leaflets are gradually replaced by the Internet [32]. Moreover, the number of people receiving health education through the Internet increased significantly. China is a big Internet country, and as of June 2023, the number of Internet users in China had reached 1.079 billion, an increase of 11.09 million from December 2022 [33]. Due to the characteristics of fast information transmission, many Internet users, and many platform choices, the Internet is gradually becoming an important way of health education [34]. Therefore, the traditional audio-visual media and the Internet will be the main way to carry out TB health education in the future. Before and now, Chinese people preferred illustrated and audio-visual TB advertising materials, which conveyed more intuitive and easier to understand information, and previous studies mentioned that people who received tuberculosis advertising through these two kinds of materials learned better and had a more comprehensive understanding of tuberculosis [35], which proved that illustrated and audio-visual materials are more suitable as advertising materials for TB. Therefore, when carrying out TB health education, TB prevention and control institutions in China should choose more graphic and audio-visual materials to promote on traditional audio-visual media and the Internet.

Before and now, We-Medias and search engines were the main sources for Chinese people to answer health-related questions on the Internet. In 2023, 78.0% of Internet users used search engines such as Baidu and Google, and 97.1% used We-Medias such as WeChat and Weibo, so it was more convenient for people to answer health-related questions through search engines and We-Media [33]. Similar to TB advertising materials, Chinese people preferred to use illustration and short-form video to answer health-related questions, which can make Chinese people more receptive to new knowledge [36, 37]. Therefore, when TB prevention and control institutions in China publish health-related questions in the future, health-related questions should be published in short-form videos and illustrated forms in the We-Media and search engines.

### Strengths and limitations

Compared with previous surveys, the study covers people from many provinces in eastern, central and western China, and the large sample size ensures the representativeness of the study. Moreover, this study is the latest national TB health promotion study in China, and the last one was the 13th Five-Year Plan. The questionnaire of this study was based on the 13th Five-Year Plan questionnaire, which was discussed by many experts. The occupation was subdivided, and the items for answering health-related questions were added. The people who collected information in this study were all from county (district) tuberculosis prevention and control institutions and had a good understanding of TB. They were supervised by provincial and municipal tuberculosis prevention and control institutions. This study also has some limitations. This study may have produced non-response bias when collecting data, but the large number of participants and large sample size may alleviate the limitation. The implementation time of End TB Action Plan is 2019–2022, and the implementation time of the 13th Five-Year Plan is 2016–2020, with two overlapping years, which may have some influence on the evaluation results.

## Conclusions

In order to end TB in China, it is essential to raise awareness of TB among the Chinese people. According to the results of this study, TB health promotion action played a positive role in China, but the awareness that TB is a chronic infectious disease and that the vast majority of patients can be cured was inadequate, so it is necessary to strengthen TB health education for those people. People of the elderly, primary school and below, and retirement had poor awareness of the TB core information. People of the elderly, urban residence, primary school and below, and equipment operators received less TB health education. It is necessary to make more use of the Internet to carry out TB health education with audio-visual and illustrated materials. Health-related questions knowledge should be published in short-form video and illustrated forms in the We-Media and search engine more.

## Data Availability

All data generated and analyzed during this study are included in this published article.

## Abbreviations

TB: Tuberculosis
TV: Television
COR: Crude Odds Ratio
AOR: Adjust Odds Ratio
CI: Confidence Interval
